# Effect of fatigability on sprint time performance and force-velocity profile according to maturity status in young rugby players

**DOI:** 10.1371/journal.pone.0316947

**Published:** 2025-01-15

**Authors:** Paul Galantine, Anthony Sudlow, Arnaud Hays, Freddy Maso, Pascale Duché, Denis Bertin

**Affiliations:** 1 Centre National de la Recherche Scientifique, Institut des Sciences du Mouvement, Aix-Marseille Univ, Marseille, France; 2 Jeunesse-Activité Physique et Sportive-Santé, Université de Toulon, Toulon, France; 3 Health Improvement through Physical Exercise Human Lab, Centre National de la Recherche Scientifique, Institut Paoli-Calmettes, Aix-Marseille Univ, Université de Toulon, Marseille, France; 4 Montferrand Sports Association, Clermont Ferrand, France; University of Potsdam, GERMANY

## Abstract

Little is known about the influence of fatigue in repeated overground sprinting on force-velocity properties in children and adolescents, while this ability to repeat sprints is important for future progress in rugby union. Sprint time decline is commonly used to assess fatigability. However, it does not provide data on biomechanical aspects of sprint performance such as maximal power, force, and velocity production. As sprint time performance and force-velocity properties do not linearly change during adolescence, considering maturity status is important. This study aimed to assess the effect of fatigue on sprint time performance fatigability, force-velocity parameters, and mechanical effectiveness according to maturity status. A group of fifteen boys (12.5 ± 0.5 years) children and a group of seventeen boys (15.1 ± 0.6 years) adolescent rugby players completed seven blocks, consisting of a 30-meter sprint followed by five minutes of high-intensity exercise with one minute of passive recovery. The force-velocity parameters were calculated at each sprint, and performance decrement was assessed using a fatigue index. A main effect of block repetition was found for maximal power output, maximal force, maximal velocity, 30-meter sprint time, fatigue index and mechanical effectiveness parameters with large effect sizes (p <0.001; $\eta_{p}^{2}$ = 0.19 to 0.47) and without a main effect of maturity status (p = 0.37 to 0.99; $\eta_{p}^{2}$ = 0.00 to 0.05). This could be explained by the modalities (duration, intensity, recovery) of the protocol and the training level of the adolescent group. For both groups, the decrease in maximal power output was due more to a reduction in maximal velocity than force, and mechanical effectiveness was negatively impacted. Coaches could prioritize the training of horizontal force at high velocity under fatigue conditions, as this ability tends to be the most affected. They could also incorporate training on mechanical effectiveness as this is a determinant in team sports.

## Introduction

In rugby union, repeatedly sprinting is a key physical attribute, both in training and in matches. Indeed, players are often exposed to repeated high-intensity activities (running, tackling, rucks, mauls and sprinting) interspersed with short periods of low-intensity activity (jogging and walking) [1]. The ability to maintain a high level of performance over repeated high-intensity efforts of short duration interspersed with short recovery periods is a key factor in rugby union [1]. Previous studies that have assessed this ability to repeat high-intensity efforts in team sports have mainly focused on sprint repetition [2–4]. However, an effort that incorporates several high-intensity actions interspersed with periods of low-intensity and recovery would be more representative of the efforts encountered by players during training and matches [5]. In general, impaired sprint time has been assessed as the main indicator of fatigue [6]. However, sprint time alone does not offer insights into the underlying biomechanical aspects of force and velocity production. These abilities can be described through the linear force-velocity (F-v) relationship, which is characterized by theoretical maximal horizontal force (*F*_0_) and theoretical maximal horizontal velocity (*v*_0_) [7]. Both characterize the ability to produce high levels of horizontal force at low and high velocity.

There has been a growing focus on exercise-induced neuromuscular fatigability in children and adolescents [8, 9]. The term ‘children’ will be used to designate individuals who have not yet experienced their peak height velocity (pre-PHV), and the term ‘adolescents’ will be used to designate individuals who have already experienced their PHV (post-PHV). This increased attention can be attributed, in part, to the growing participation of children and adolescents in high-level sports, where they are frequently exposed to demanding training regimens comparable to those of adults [8]. Maximal and supra-maximal intensity exercise, either using a treadmill or cycle ergometry, has been used to investigate fatigue and fatigability in children and adolescents [10–12]. Fatigability has been defined as a decline in an objective measure of performance (sprint time, for example) during and/or after a given exercise [9, 13]. It has been well-established that children have a lower fatigability than adolescents when performing whole-body dynamic activities, such as maximal cycling or short running bouts under laboratory or field conditions [9–12]. Consequently, repeated sprint ability is higher in children compared to adolescents.

To date, four studies in adults but only one study in adolescents have directly investigated the influence of fatigue (repeated overground sprinting) in team sports on force-velocity-power properties under field conditions [2–6]. These studies showed that sprint time performance decreased and *v*_0_ was mainly impacted relative to *F*_0_. Parameters characterizing the mechanical effectiveness of force application such as the ability to orient force horizontally at low velocities (*RF*_max_) and the ability to limit the loss of this ability with increasing running velocity (*D*_RF_) [14] were also impacted [2, 3, 15]. To our knowledge, no study has assessed the effect of fatigue according to maturity status on a sprint repetition task with a specific focus on the F-v parameters in field conditions. Yet, considerable changes associated with growth and maturity status are likely to influence force, velocity, and power production. For instance, the increase in muscle cross-sectional area during maturation is likely to influence the force component, while longer sarcomeres may enhance velocity capabilities [16–18]. Additionally, factors like motor unit recruitment can affect both aspects of the F-v relationship, which contributes to maximal power output (*P*_max_) [17]. Studies have also shown that this ability to voluntarily activate a greater percentage of motor units increases with maturity status [19, 20]. As physical performance does not linearly change during the critical stage of adolescence [21], considering maturity status is important.

Furthermore, it is known that the mechanisms of neuromuscular fatigability differ according to maturity status given that adolescents have greater fatigability than children during high-intensity intermittent exercise [8, 9]. Indeed, young children may cope better with shorter rest periods commonly used by adolescents during maximal-intensity intermittent training. Consequently, the selection of training parameters (such as duration, intensity, and recovery) may not only be based on training goals but also adjusted according to maturity status [22]. This knowledge could contribute to effectively managing training load and recovery in maturing athletes [8]. This study aimed to assess the effect of fatigue on sprint time performance fatigability, F-v parameters, and mechanical effectiveness according to maturity status. The hypotheses were as follows: (i) sprint time performance and *P*_max_ decrease following the fatiguing task for all groups with a smaller decrement for children than for adolescents; (ii) There is a greater decrease in *v*_0_ than in *F*_0_ for both children and adolescents; (iii) Mechanical effectiveness (*RF*_max_ and *D*_RF_) will be impacted by fatigue, induced by the fatiguing task, in both children and adolescents.

## Methods

### Participants

A power analysis was conducted before the study (G*Power 3) using the following test details: “ANOVA: repeated measures, within-between interaction”, an effect size of 0.25, alpha of 0.05 and power of 0.8, which suggested the total sample size of the study should include 18 subjects. Using a conversion formula $\left(\eta_{p}^{2}=\frac{f2}{1}+f2\right)$, an effect size f of 0.25 gives a partial eta squared of 0.06, which is a medium effect. Fifteen boys (age: 12.5 ± 0.5 years) children and seventeen boys (age: 15.1 ± 0.6 years) adolescent rugby players participated in this study. Adolescent players were recruited at the training centre of Association Sportive Montferrandaise (ASM, Clermont Auvergne Rugby, France) and played at the highest level for their age and category. The children trained three to four times a week (plus one match) and the adolescents group trained six times a week (plus one match). For both groups, the training includes both technical training and physical conditioning (sprint training, speed endurance training and high-intensity aerobic training). Training sessions usually lasted between 60 and 120 minutes. The training experience of the children and adolescents groups was 6.5 ± 1.7 years and 9.2 ± 1.8 years of rugby, respectively. Inclusion criteria were male, age 12–17 years (included), engaged in competitive rugby and physically able to perform the tests. In addition, the medical staff had to agree for each player to take part in the study. Exclusion criteria included previous surgery on the lower limbs or injuries identified within the three months preceding the tests. Each participant was informed about the objectives, protocol, and tests of the study. They gave their written informed consent to participate in the research, and written consent was also obtained from parents or legal guardians. The protocol was approved by the national ethical committee of Sport Sciences (CERSTAPS n° IRB00012476-2022-14-09-197).

### Study design

**Fig 1:**
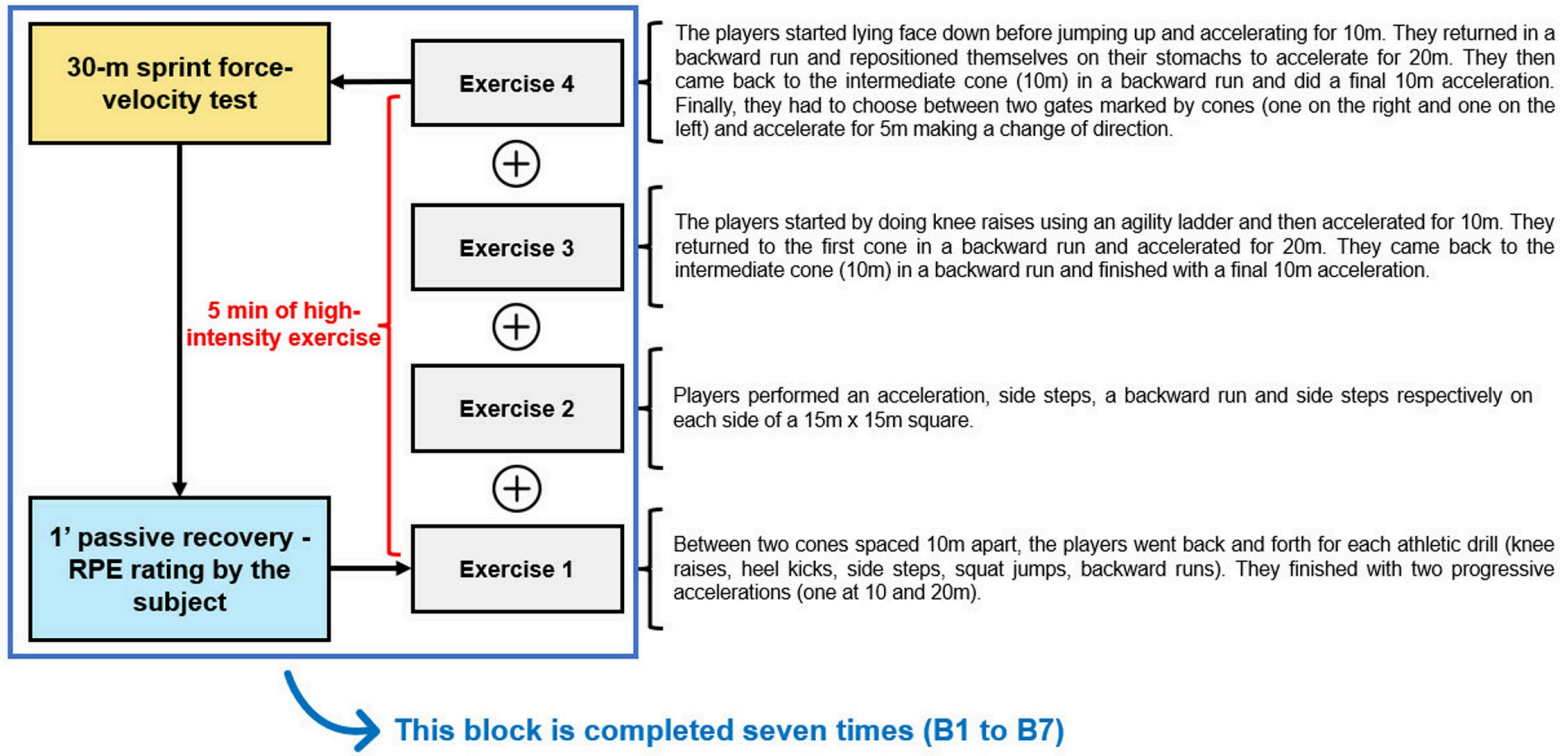
Experimental protocol for the fatiguing task.

The experimental design of the study took place over the sporting season from August 2022 to June 2023. The adolescents were recruited in September (from 15/09/2022 to 18/09/2022) and completed the protocol from 19/09/2022 to 23/09/2022 (no competitive matches). The children were recruited in October (from 12/10/2022 to 20/10/2022) and completed the protocol from 24/10/2022 to 28/10/2022 (training camp without competitive matches). All participants were tested in two experimental sessions separated by at least 24 hours. During the first experimental session, anthropometric characteristics and maturity status were assessed. Then, they performed two 30-meter sprints after a standardized warm-up (five minutes of low-intensity running, several sprint-specific drills, and active-dynamic stretching for both groups) to represent their baseline sprint time performance. In the second experimental session and after a standardized 15-minute warm-up (same warm-up as the first session plus a trial block to test the protocol), participants performed seven blocks consisting of one 30-meter sprint and five minutes of high-intensity exercise (accelerations, decelerations, changes of direction) with one minute of passive recovery (Fig 1). The players performed the second experimental session in groups of two to four. Participants were equipped with a Polar Pro chest strap with a Polar H10 heart rate (HR) sensor attached over the sternum (Polar Electro Oy, Kempele, Finland), and their rating of perception of effort (RPE) and internal training load was assessed to monitor the intensity of effort after each block.

### Measurements

#### First experimental session

*Anthropometry and maturity status*. Stature and sitting height were determined using a stadiometer (0.01 m, The Leicester Height Measure, Tanita Corp., Japan) and body mass was obtained using a clinical scale (0.1 kg, Tanita UM-076, Tanita Corp., Japan). To measure sitting height, the participants sat on the support of the stadiometer with their legs straight, their backs straight and their gaze fixed. Estimated maturity status was measured through somatic maturation derived from the estimated age at peak height velocity (PHV), using the algorithm proposed by Mirwald et al. [23]. This method estimates distance in years from PHV through anthropometric variables (stature, sitting height, body mass) and date of birth. The prediction of age at PHV was determined by subtracting the maturity offset from chronological age. Groups were defined as children (pre-PHV): ≤ - 0.5 and adolescents (post-PHV): ≥ + 0.5 [24].

*Baseline sprint time performance*. Participants performed two maximal 30-meter sprints, from which the best sprint time was retained. This best sprint time was used as a criterion score to be compared with the first sprint time performed during the second experimental session (B1). Indeed, to prevent pacing effects occurring during such protocols [25], participants were requested to achieve at least 95% of their respective criterion score during the first sprint of the second experimental session (B1). A single system of timing gates (Witty Microgate, Microgate, Bolzano, Italy) was set one meter apart at 0 and 30 meters and was positioned approximately at the level of the participant’s hips. In addition, relative and absolute reliability was assessed using the intraclass correlation coefficient (ICC) and coefficient of variation (CV), respectively.

#### Second experimental session

*Fatiguing task*. After a standardized 15-minute warm-up, participants performed seven blocks of five minutes of high-intensity exercise, each followed by a 30-meter sprint and one minute of passive recovery (Fig 1). The players started the protocol with a sprint, and the whole task took about 35–40 minutes. The experimenters supervised and encouraged the players through each block. If a player did not have time to complete all the exercises in five minutes, they were directed to the 30-meter sprint, regardless of their progress. If a player finished all the exercises in less than five minutes, they were asked to continue with exercise four until the end of the time limit. An experimenter was responsible for monitoring the time to ensure the task was isotime for each player.

*F-v sprint profile*. Participants completed a 30-meter sprint at maximum speed at each block (B1 to B7) on a synthetic outdoor field. During the tests, the experimenter signalled the start of each sprint using verbal instructions. Each subject wore their usual rugby boots and set off from a standing start to limit movements that could disrupt data acquisition. The raw velocity-time data were measured using a radar (Stalker ATS II, Applied Concepts, USA) at 46.875 Hz. The radar was positioned five meters behind the starting line on a tripod and adjusted to the height of the subject’s hips.

Raw velocity-time data for each acceleration were manually processed in the software package commercially provided with the radar to remove any aberrant data points (STATS, Version 5.0.3.0, Applied Concepts, USA). Subsequently, the horizontal velocity-time curve for each player was modelled using a mono-exponential function via a custom-made computer program (VBA, Microsoft Excel, USA). A macroscopic inverse dynamic approach applied to the centre of mass [14] was then used to estimate the relevant step-averaged parameters of the F-v-power relationship: *F*_0_, *v*_0_, *P*_max_ (determined as the apex of the power-velocity relationship), and the F-v profile (*S*_FV_). Air temperature, atmospheric pressure and wind speed were measured using a weather station (Bresser, Germany) and then used to estimate air density and friction force during the sprint [26]. Parameters characterizing the mechanical effectiveness (*RF*_max_ and *D*_RF_) were determined [14].

*Fatigue index*. The decrement in performance was assessed using a fatigue index (FI) computed from 30-meter times during each sprint [27]. FI accounting for percent decrement in performance was computed as the percent difference between the best sprint time performed during the second experimental session (number n of repetitions multiplied by the best sprint time over n), and the actual sprint time performed (sum of sprint time values performed over n):

FI_n_ = 100 – (100 * [sum of sprint time values for sprints 1 to n]) / [best sprint time * n])

With best sprint time defined as the best time run during sprints 1 to n thorough the second experimental session.

*Heart rate monitor*. The HR was recorded continuously with a Polar H10 HR sensor (Polar Electro Oy, Kempele, Finland) attached to a chest strap. The HRmean was calculated for each block from the lowest HR point after the minute of passive recovery to the end of the fatiguing task (i.e. just before the sprint).The maximum HR was estimated using the formula of Tanaka et al. (2001) [28] (209–0.7 x age). Based on this, high-intensity was defined as an effort between 80 and 100% of maximum HR [29].

*RPE and internal training load*. Players recorded their RPE using the CR-10 Borg’s scale which has been widely used to estimate the perceived exertion of a session for children and adolescents [30, 31]. The players had to mark the number corresponding to their RPE at the end of each sprint. All participants were familiar with this method as it was frequently used during training before the start of the study. The internal training load of each block was calculated by multiplying the RPE score by five minutes [32]. The validity, reliability and internal consistency of the session-RPE method have been demonstrated in several sports with children and adolescents among various expertise levels [33, 34].

### Statistical analyses

Data distributions were assessed using the Shapiro-Wilk normality test, and the homogeneity of variances was assessed by the Levene test. All data are presented as mean ± SD. To compare anthropometric parameters, somatic maturation and training experience between children and adolescents, independent-sample t-tests (two-tailed) were used. To compare *F*_0_ and *v*_0_ decrements between B1 and B7 for both groups, an independent-sample t-test (one-tailed) was used. If assumption of normality and variances were not met, the Mann-Whitney test was used. The standardized mean difference (SMD) (Hedges’s *g*) was calculated with thresholds of 0.2, 0.5, and 0.8 defined as small, moderate, and large effects, respectively [35]. For variables that did not meet the conditions of application, the formula of Rosenthal [36] was used to calculate the effect size: r = z/√N where z is the test statistic and N is the number of observations. For the 30-metre sprint time, F-v and mechanical effectiveness parameters, the values in the first block (B1) were considered to represent 100% of the players’ abilities. All values for blocks B2 to B7 were expressed as a percentage change from B1 (100%). Statistical tests were performed on these percentage values and not on the absolute values. The thresholds for interpreting relative reliability results were: 0.20–0.49, 0.50–0.74, 0.75–0.89, 0.90–0.98, and > 0.99 for low, moderate, high, very high, and extremely high, respectively [37]. Absolute reliability was considered acceptable if the CV was ≤ 10%. The average reliability of sprint time was interpreted as acceptable for an ICC ≥ 0.75 and a CV ≤ 10%, moderate when ICC < 0.75 or CV > 10%, and poor when ICC < 0.75 and CV > 10% [37]. To analyse the effect of fatigability on the F-v profile, sprint time performance, and mechanical effectiveness according to maturity status (pre- and post-PHV) and block (B1 to B7), a mixed two-way repeated measures analysis of variance (ANOVA) was performed. Each ANOVA was performed after checking for distribution normality with Shapiro-Wilk’s test and sphericity of variance with Mauchly’s test. When an ANOVA revealed a main block or interaction effect, pairwise comparisons using Bonferroni post hoc tests were used to determine exactly the differences between the means of the different groups at each block. Partial eta-squared ($\eta_{p}^{2}$) was used for effect size calculations in ANOVA. The effect sizes are considered small, medium, and large effects if the calculated $\eta_{p}^{2}$ are approximately equal to 0.01, 0.06, and 0.14, respectively. Statistical analyses were performed using SPSS (IBM SPSS, v26.0). The α risk was set at 5%, *i*.*e*., p < 0.05.

## Results

### Anthropometry and maturity status

Table 1 presents descriptive data and group comparisons between children and adolescents for anthropometric characteristics, somatic maturation, and training experience. All the parameters were higher for adolescents than for children (p < 0.001; SMD = -0.6 to 5.3).

**Table 1:** Comparisons of anthropometry, somatic maturation, and training experience between children and adolescents.

Variables *(mean ± SD)*	Children (n = 15)	Adolescents (n = 17)	*p* value	t	SMD
BM (kg)	46.2 ± 8.6	75.3 ± 8.9	**< 0.001**	9.1	3.3
Stature (cm)	155.2 ± 8.3	178.9 ± 7.0	**< 0.001**	8.5	3.1
BMI (kg·m^-²^)	19.1 ± 2.5	23.5 ± 2.0	**< 0.001**	5.5	2.0
SH (cm)	76.7 ± 3.7	90.2 ± 3.7	**< 0.001**	-4.8	-0.9
Maturity-Offset (years)	-1.7 ± 0.6	+1.5 ± 0.6	**< 0.001**	14.4	5.3
Training experience (years)	6.5 ± 1.7	9.2 ± 1.8	**< 0.001**	-3.5	-0.6

*SMD*, standardized mean difference; *t*, test statistic*; BM*, body mass; *BMI*, body mass index; *SH*, sitting height.

### F-v parameters

The 95% criterion score for the first sprint performed during the second experimental session (B1) was almost completely satisfied (mean ± SD: 96.7 ± 2.5%). Relative and absolute reliability between baseline sprint time and 30-meter sprint time at B1 was very high (ICC = 0.98) and acceptable (CV = 1.7%) allowing acceptable average reliability (ICC ≥ 0.75 and a CV ≤ 10%).

**Fig 2:**
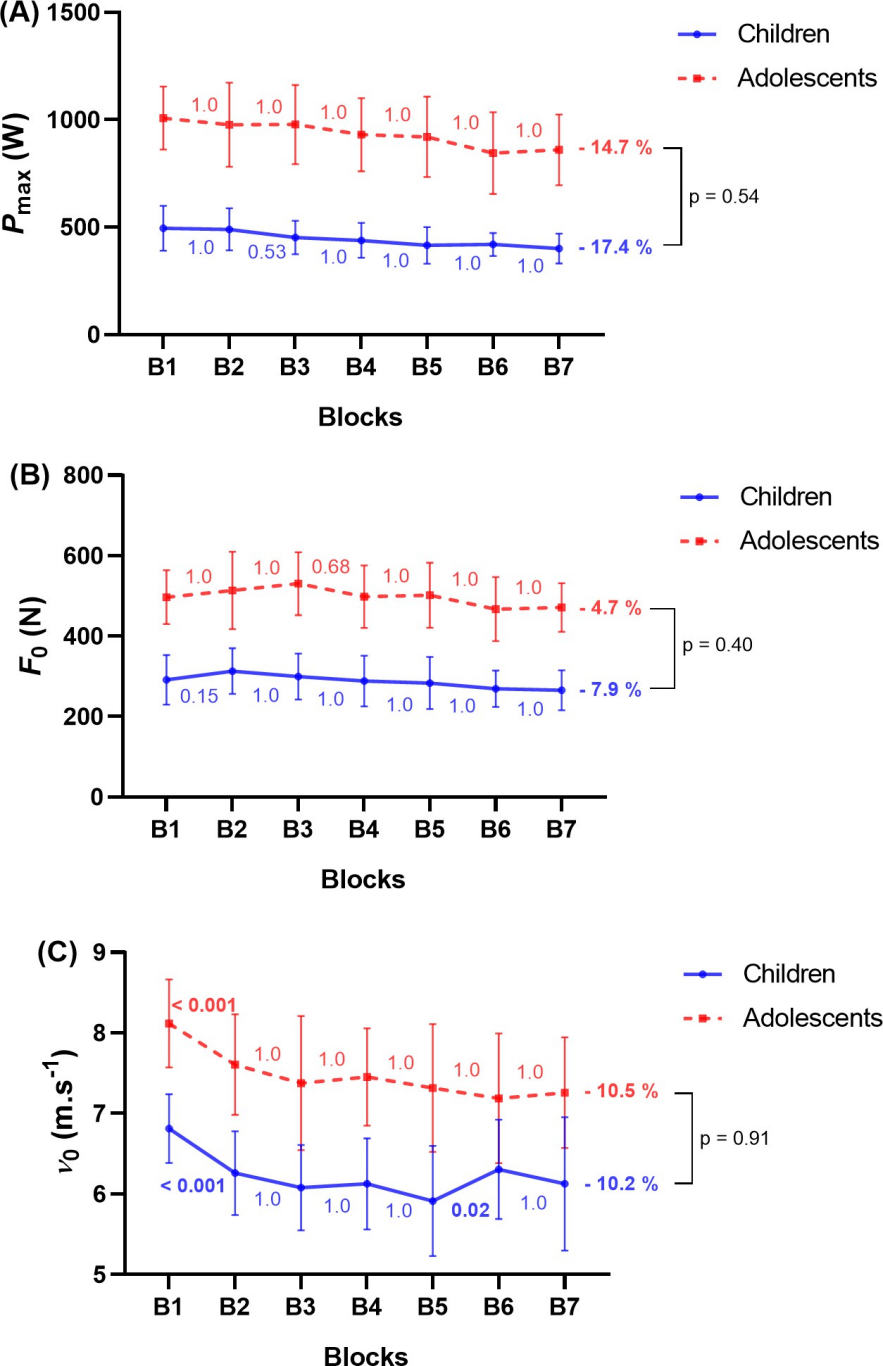
Mixed two-way repeated measures analysis of variance for maximal power output (*P*_max_, (A)), theoretical maximal force (*F*_0_, (B)), and theoretical maximal velocity (*v*_0_, (C)) between children (blue solid line) and adolescents (red dotted line). Post hoc results for the main block effect are represented for children and adolescents between each block. The significance for each group concerning the final decrement is represented (B1 vs B7).

Table 2 presents descriptive data and mixed two-way repeated measures ANOVA for F-v parameters between children and adolescents. There was a small interaction effect for all the F-v variables (p = 0.16 to 0.66) and all the effect sizes were small ($\eta_{p}^{2}$ = 0.02 to 0.05). A main effect of block repetition was found for *P*_max_, *F*_0_, *v*_0_ and *S*_FV_ relative to body mass with large effect sizes (p < 0.001; $\eta_{p}^{2}$ = 0.19 to 0.37) and without a main effect of maturity status. In children, *P*_max_ decreased by 17.4% from the first sprint (495 ± 101 W) to the last sprint (401 ± 67 W, p < 0.001, Fig 2), *F*_0_ by 7.9% from the first sprint (291 ± 59 N) to the last sprint (265 ± 48 N, p = 0.17, Fig 2), *v*_0_ by 10.2% from the first sprint (6.8 ± 0.4 m.s^-1^) to the last sprint (6.1 ± 0.8 m.s^-1^, p = 0.001, Fig 2) and *S*_FV_ relative to body mass increased by 4.3% from the first sprint (0.93 ± 0.1 N.s.m^-1^.kg^-1^) to the last sprint (0.97 ± 0.2 N.s.m^-1^.kg^-1^, p = 1.0). In adolescents, *P*_max_ decreased significantly by 14.7% from the first sprint (1008 ± 142 W) to the last sprint (860 ± 160 W, p < 0.001, Fig 2), *F*_0_ by 4.7% from the first sprint (497 ± 65 N) to the last sprint (471 ± 59 N, p = 1.0, Fig 2), *v*_0_ by 10.5% from the first sprint (8.1 ± 0.5 m.s^-1^) to the last sprint (7.3 ± 0.7 m.s^-1^, p < 0.001, Fig 2) and *S*_FV_ relative to body mass increased by 7.2% from the first sprint (0.82 ± 0.1 N.s.m^-1^.kg^-1^) to the last sprint (0.87 ± 0.1 N.s.m^-1^.kg^-1^, p = 1.0). The degrees of freedom and test statistics for each variable are presented in S1 Table. In both groups combined, the decrement of *v*_0_ was greater (p = 0.04; SMD = -0.2; test statistic = -1.8) than that of *F*_0_ at the end of the fatiguing protocol (-10.4 ± 8.5% vs -6.2 ± 10.5%, respectively).

**Table 2:** Descriptive data and mixed two-way repeated measures analysis of variance for F-v parameters between children and adolescents.

	Descriptive data *(mean ± SD)*								Block*Maturity status		Block		Maturity status	
Variables	Groups	B1	B2	B3	B4	B5	B6	B7	*p* value	$\eta_{p}^{2}$	*p* value	$\eta_{p}^{2}$	*p* value	$\eta_{p}^{2}$
*P*_max_ (%)	***pre-PHV***	100.0 ± 0.0	99.6 ± 9.2	92.6 ± 10.8	89.7 ± 12.2	85.2 ± 14.2	86.8 ± 12.2	82.6 ± 13.8	0.19	0.05	**< 0.001**	0.37	0.65	0.01
	***post-PHV***	100.0 ± 0.0	96.4 ± 9.4	97.0 ± 10.5	92.1 ± 8.0	90.9 ± 10.8	83.2 ± 11.9	85.3 ± 10.3						
*F*_0_ (%)	***pre-PHV***	100.0 ± 0.0	108.3 ± 9.0	104.0 ± 13.5	99.9 ± 13.3	98.1 ± 15.1	94.0 ± 13.3	92.1 ± 11.9	0.53	0.03	**< 0.001**	0.23	0.81	0.00
	***post-PHV***	100.0 ± 0.0	103.2 ± 12.2	107.0 ± 9.1	100.3 ± 8.7	100.9 ± 8.9	94.1 ± 11.8	95.3 ± 8.8						
*v*_0_ (%)	***pre-PHV***	100.0 ± 0.0	92.0 ± 4.6	89.3 ± 5.0	90.0 ± 5.2	87.0 ± 8.5	92.7 ± 7.2	89.8 ± 10.1	0.16	0.05	**< 0.001**	0.37	0.74	0.00
	***post-PHV***	100.0 ± 0.0	93.7 ± 5.0	90.7 ± 7.4	92.0 ± 5.6	90.2 ± 7.9	88.5 ± 7.9	89.5 ± 6.7						
*S*_FV_ (N·s·m^-1^·kg^-1^)	***pre-PHV***	0.93 ± 0.1	1.09 ± 0.1	1.08 ± 0.2	1.04 ± 0.2	1.05 ± 0.2	0.95 ± 0.2	0.97 ± 0.2	0.66	0.02	**< 0.001**	0.19	0.97	0.00
	***post-PHV***	0.82 ± 0.1	0.91 ± 0.2	0.97 ± 0.2	0.90 ± 0.1	0.92 ± 0.1	0.87 ± 0.2	0.87 ± 0.1						
*S*_FV_ (%)	***pre-PHV***	100.0 ± 0.0	118.1 ± 12.2	117.1 ± 19.0	111.6 ± 17.3	114.2 ± 23.4	102.5 ± 19.4	104.3 ± 20.3						
	***post-PHV***	100.0 ± 0.0	110.8 ± 18.0	118.8 ± 15.1	109.6 ± 13.9	112.7 ± 14.2	107.4 ± 18.3	107.2 ± 13.6						

*pre-PHV*, children; *post-PHV*, adolescents; *P*_max_, maximal power output; *F*_0_, theoretical maximal force; *v*_0_, theoretical maximum velocity; *S*_FV_, force-velocity slope relative to body mass.

### Mechanical effectiveness, sprint time performance, RPE, internal training load, FI and HRmean

**Fig 3:**
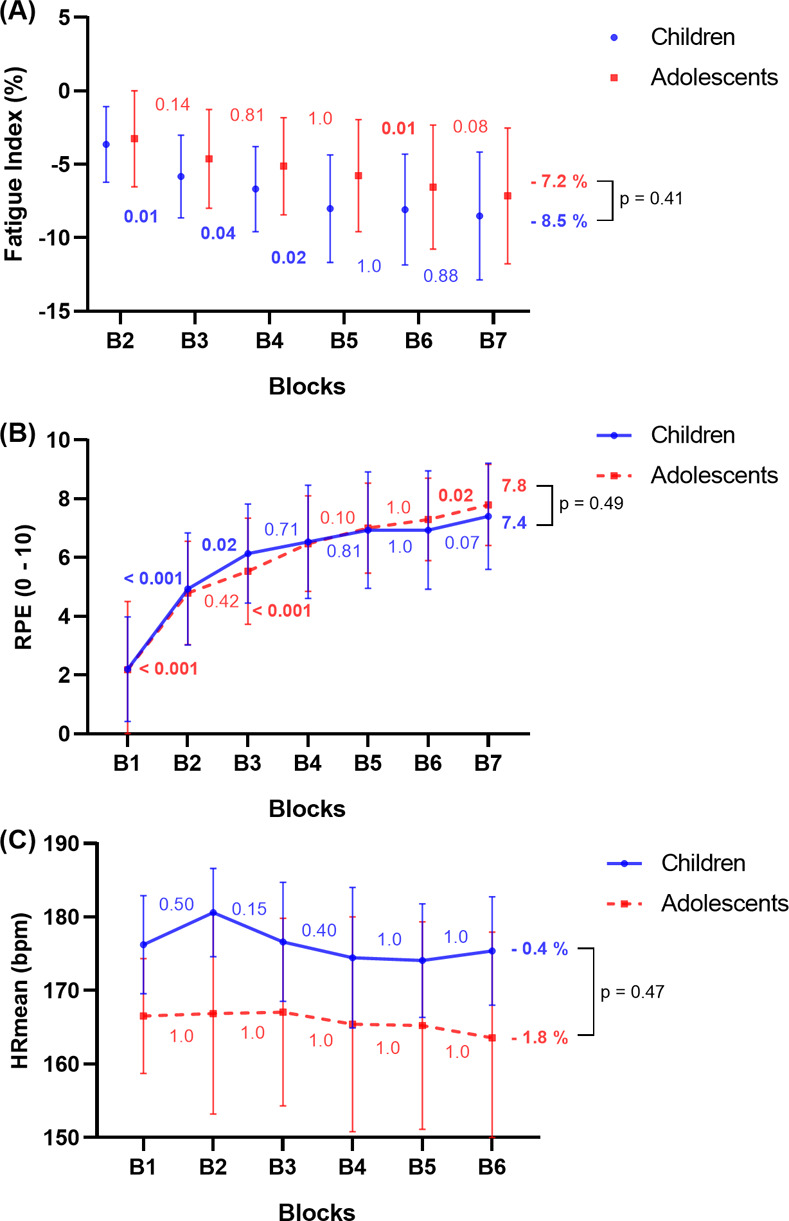
Mixed two-way repeated measures analysis of variance for Fatigue Index (A), rating of perceived exertion (RPE, (B)), and mean heart rate (HRmean, (C)) between children (blue solid line) and adolescents (red dotted line). Post hoc results for the main block effect are represented for children and adolescents between each block. The significance for each group concerning the final decrement is represented (B1 vs B7).

Table 3 presents descriptive data and mixed two-way repeated measures ANOVA for mechanical effectiveness, sprint time performance, RPE and FI between children and adolescents. There was a small interaction effect for all the variables (p = 0.14 to 0.63) with small to moderate effect sizes ($\eta_{p}^{2}$ = 0.02 to 0.06). A main effect of block repetition was found for *RF*_max_, *D*_RF_, 30-meter sprint time, RPE (and therefore internal training load) and FI with large effect sizes (p < 0.001; $\eta_{p}^{2}$ = 0.19 to 0.47) and without a main effect of maturity status. In children, *RF*_max_ decreased by 7.9% from the first sprint (43.1 ± 2.8%) to the last sprint (39.7 ± 4.1%, p = 0.01), *D*_RF_ increased by 5.5% from the first sprint (-8.8 ± 0.8%.s.m^-1^) to the last sprint (-9.2 ± 1.6%.s.m^-1^, p = 1.0) and 30-meter sprint time by 10.9% from the first sprint (5.18 ± 0.22 s) to the last sprint (5.75 ± 0.58 s, p < 0.001). In adolescents, *RF*_max_ decreased by 5.2% from the first sprint (46.0 ± 3.1%) to the last sprint (43.5 ± 3.4%, p = 0.05), *D*_RF_ increased by 8.5% from the first sprint (-7.6 ± 0.9%.s.m^-1^) to the last sprint (-8.2 ± 0.9%.s.m^-1^, p = 1.0) and 30-meter sprint time by 10.5% from the first sprint (4.60 ± 0.28 s) to the last sprint (5.09 ± 0.53 s, p < 0.001). The degrees of freedom and test statistics for each variable are presented in S1 Table. For HRmean, there were no differences between consecutive blocks for the children and adolescents groups (Fig 3).

**Table 3:** Descriptive data and mixed two-way repeated measures analysis of variance for mechanical effectiveness parameters, sprint time performance, RPE and FI between children and adolescents.

	Descriptive data *(mean ± SD)*								Block*Maturity status		Block		Maturity status	
Variables	Groups	*p* value	$\eta_{p}^{2}$	*p* value	*p* value	$\eta_{p}^{2}$	*p* value	B7	*p* value	$\eta_{p}^{2}$	*p* value	$\eta_{p}^{2}$	*p* value	$\eta_{p}^{2}$
*RF*_max_ (%)	***pre-PHV***	43.1 ± 2.8	43.9 ± 2.5	42.5 ± 3.1	41.6 ± 4.6	40.7 ± 4.7	40.5 ± 3.4	39.7 ± 4.1	0.23	0.04	**< 0.001**	0.29	0.52	0.01
	***post-PHV***	46.0 ± 3.1	45.9 ± 4.4	46.5 ± 4.0	45.1 ± 4.0	45.0 ± 4.5	43.0 ± 4.9	43.5 ± 3.4						
*RF*_max_ (%)	***pre-PHV***	100.0 ± 0.0	102.1 ± 4.8	98.7 ± 6.1	96.6 ± 7.5	94.5 ± 8.6	94.1 ± 7.9	92.1 ± 7.4						
	***post-PHV***	100.0 ± 0.0	99.7 ± 6.0	101.1 ± 4.7	98.0 ± 4.7	97.8 ± 5.5	93.4 ± 7.2	94.8 ± 5.3						
*D*_RF_ (%·s·m^-1^)	***pre-PHV***	-8.8 ± 0.8	-10.3 ± 1.0	-10.3 ± 1.4	-9.9 ± 1.6	-10.1 ± 1.7	-9.0 ± 1.4	-9.2 ± 1.6	0.63	0.02	**< 0.001**	0.19	0.96	0.00
	***post-PHV***	-7.6 ± 0.9	-8.4 ± 1.5	-9.1 ± 1.6	-8.4 ± 1.3	-8.6 ± 1.3	-8.2 ± 1.4	-8.2 ± 0.9						
*D*_RF_ (%)	***pre-PHV***	100.0 ± 0.0	118.0 ± 11.8	117.5 ± 18.4	112.2 ± 16.9	115.2 ± 23.3	103.4 ± 19.2	105.5 ± 20.7						
	***post-PHV***	100.0 ± 0.0	110.9 ± 17.5	118.9 ± 15.0	110.2 ± 13.8	113.4 ± 14.4	108.8 ± 18.5	108.5 ± 13.7						
30-meter sprint time (s)	***pre-PHV***	5.18 ± 0.22	5.56 ± 0.39	5.71 ± 0.42	5.66 ± 0.38	5.87 ± 0.55	5.60 ± 0.38	5.75 ± 0.58	0.14	0.06	**< 0.001**	0.37	0.37	0.03
	***post-PHV***	4.60 ± 0.28	4.88 ± 0.38	4.92 ± 0.52	4.89 ± 0.40	4.98 ± 0.52	5.07 ± 0.52	5.09 ± 0.53						
30-meter sprint time (%)	***pre-PHV***	100.0 ± 0.0	107.1 ± 5.1	110.1 ± 4.9	109.2 ± 4.5	113.2 ± 9.3	108.0 ± 6.3	110.9 ± 9.4						
	***post-PHV***	100.0 ± 0.0	106.0 ± 6.8	106.8 ± 6.9	106.3 ± 5.1	108.0 ± 7.6	110.1 ± 7.7	110.5 ± 7.8						
RPE	***pre-PHV***	2.2 ± 1.7	4.9 ± 1.8	6.1 ± 1.6	6.5 ± 1.9	6.9 ± 1.9	6.9 ± 1.9	7.4 ± 1.7	0.41	0.03	**< 0.001**	0.79	0.99	0.00
	***post-PHV***	2.2 ± 2.3	4.8 ± 1.7	5.5 ± 1.8	6.5 ± 1.6	7.0 ± 1.5	7.3 ± 1.4	7.8 ± 1.3						
Internal training load (a.u)	***pre-PHV***	11.0 ± 8.6	24.7 ± 9.2	30.7 ± 8.1	32.7 ± 9.3	34.7 ± 9.6	34.7 ± 9.7	37.0 ± 8.7	na				na	
	***post-PHV***	10.9 ± 11.3	24.0 ± 8.5	27.6 ± 8.8	32.4 ± 7.9	35.0 ± 7.4	36.5 ± 6.8	39.0 ± 6.7						
FI (%)	***pre-PHV***	na	-3.6 ± 2.5	-5.8 ± 2.7	-6.7 ± 2.8	-8.0 ± 3.5	-8.1 ± 3.6	-8.5 ± 4.2	0.36	0.03	**< 0.001**	0.47	0.24	0.05
	***post-PHV***	na	-3.3 ± 3.2	-4.6 ± 3.3	-5.1 ± 3.2	-5.8 ± 3.7	-6.5 ± 4.1	-7.2 ± 4.5						

*pre-PHV*, children; *post-PHV*, adolescents; *RF*_max_, maximum value of ratio of force; *D*_RF_, decrement in ratio of force; RPE, rating of perceived exertion; *FI*, fatigue index; na, not applicable; a.u, arbitrary units.

## Discussion

This study assessed the impact of fatigue, induced by a fatiguing task in ecological conditions, on sprint time performance fatigability and F-v-power properties in children and adolescent athletes. The main results of this study showed no effect of maturity status on fatigability, induced by fatiguing task repetition. Sprint time performance and *P*_max_ were impacted by the fatiguing protocol, confirming our first hypothesis. For both groups combined, the reduction in *P*_max_ was due more to a reduction in *v*_0_ than *F*_0_, confirming our second hypothesis. As hypothesized, the fatiguing protocol negatively impacted the mechanical effectiveness (*RF*_max_ and *D*_RF_) in both groups. In addition, all the parameters representing the intensity of the fatiguing task were not different between the two groups (HRmean, RPE and consequently training load).

### Sprint time performance and fatigability of fatiguing protocol

The results of this study do not confirm the previously reported results [9] as there was no maturity status effect on fatigability (-8.5% for children and -7.2% for adolescents). The intensity (not supra-maximal) and duration of the fatiguing task could partly explain the differences with previous research. Indeed, in most studies evaluating the effect of maturity status on the ability to repeat sprints, efforts were supra-maximal, repeated over short periods (10 to 30 seconds) with recovery periods [9] which probably did not involve aerobic metabolism as much as our protocol did. Moreover, unlike in previous studies, the participants in our study were trained athletes, which implies that both groups were used to this type of effort. Moreover, Mujika et al. [38] reported similar results with no differences in percent sprint decrement among different age groups (U11 to U18) and Sánchez-Sánchez et al. [39] reported also no differences in repeated-sprint ability decrement between U14, U16 and U18. While many studies have implemented sprint repetition protocols in running [40], the number and distance of the sprints and the nature and duration of the recovery make it difficult to compare the results. However, the generally reported performance decrement in sprinting for children and adolescents is between -2 and -5% [38, 40], which is close to our results.

Several factors could also explain why the decrement in sprint time performance was not different between the two groups. In agreement with the duration of the fatiguing task (≈ 35 to 40 minutes), the involvement of aerobic metabolism could be increasingly important to sustain the effort. For the children, our results are in line with those previously reported in the literature [9]. Their oxidative metabolic profile may have contributed significantly, in limiting the loss in performance throughout the fatiguing task. Indeed, numerous studies have indicated that a higher maximal oxygen consumption can enhance the ability to perform repeated sprints by facilitating the replenishment of phosphocreatine (PCr) and adenosine triphosphate stores during the recovery period between sprints, thus helping to maintain performance across multiple high-intensity efforts [41, 42]. For the adolescents, the duration of the effort during the fatiguing task and the passive recovery between the fatiguing task repetition (one minute) may explain why fatigability was not different from that of the children. Indeed, after a maximal six-second sprint, PCr stores can be reduced to around 35–55% of resting levels, and the complete recovery of stores requires approximately five minutes [40]. It is possible to assume that adolescents had time to resynthesize sufficient PCr (which is the most immediate reserve for the rephosphorylation of adenosine triphosphate) during exercise and passive recovery to limit their loss in performance compared to the children. In the same way, it has been reported that there is a simultaneous increase in both anaerobic capacity and oxygen kinetics during maturation [38]. These factors may have counterbalanced each other, resulting in the absence of differences in fatigability between different maturity statuses or age groups [38, 39, 43]. It should also be noted that the adolescents were more trained and had more training experience than the children (9.2 ± 1.8 years vs 6.5 ± 1.7 years, respectively). For example, high-intensity interval training, known to induce an increase in maximal oxygen consumption and mitochondrial content [44], was practiced more by adolescents. As seen previously, these physiological adaptations are involved in limiting loss in performance during sprint repetitions, especially in our protocol, where the task was of a long duration (> 30 minutes).

### F-v and mechanical effectiveness parameters

The deltas reported for *P*_max_, *v*_0_, and *F*_0_ in the present study were consistent with previous results reported [3]. In agreement with previously reported findings, *P*_max_ was altered by the fatiguing protocol with a greater decrease in *v*_0_ than *F*_0_ [2–6, 45]. Then, the ability to generate horizontal force at high speed was impacted to a greater extent than the ability to generate horizontal force at low speed for both groups combined. It has been suggested that the greater decrement in *v*_0_ rather than *F*_0_ could be due to a reduction in contraction velocity of type II fibres due to a greater impact of metabolite-induced disturbances [3]. In contrast, the smaller decline in *F*_0_ could be explained by the rapid recovery of PCr [46], enabling the initial seconds of the sprint to be less affected by fatigability [3]. The fatigue induced could also have had an impact on the hip extensors (impaired contraction capacity), which are the main muscles involved in high-velocity force production [47].

Interestingly, mechanical effectiveness (*RF*_max_ and *D*_RF_) was altered after the fatiguing protocol, confirming our third hypothesis. This is consistent with previous results as studies have already shown that mechanical effectiveness was altered after a sprint repetition protocol [2–4, 15]. These results have important implications for programming training content as the decrease in *D*_RF_ has been considered as a determinant of repeated-sprint performance in adolescents [4]. For example, for practitioners, it could be interesting to integrate work on the mechanical effectiveness parameters to improve repeated-sprint ability, which is essential in rugby, even in fatigue conditions.

### Strengths and limitations

This study provides insight into the effect of fatigability on sprint time performance and the F-v profile in field conditions according to maturity status in a trained population. This is one of the major strengths of the study in terms of its practical applications and perspectives for team sports training. Indeed, previous studies have predominantly used sprinting time, thus limiting our understanding of the mechanical determinants of power production under fatigability conditions. Some limitations of this study should be noted. Only the macroscopic power, force and velocity components of sprint performance were assessed. Therefore, it is not possible to make any definitive conclusions about the neurophysiological and metabolic mechanisms underlying the fatigability-induced changes that have been studied and discussed elsewhere [40]. Finally, it would also have been beneficial to quantify the movement characteristics of the players using a global positioning system (distance covered, accelerations, decelerations, etc.) during the fatiguing protocol to quantify better the workload performed by the players. Nevertheless, the session-RPE method used in this study is valid, reliable and useful in children and adolescents [33].

## Conclusion

This study showed that the capacity to repeat overground sprints during a fatiguing task repetition is impacted but without any maturity status effect. In accordance, children and adolescents experienced the same perceived fatigability and internal training load. For both groups, the decrease in maximal power output was due more to a reduction in maximum velocity than maximum force production. Finally, children and adolescents showed a decreased ability to orient force horizontally at low velocities (*RF*_max_) and to limit the loss of this ability with increasing running velocity (*D*_RF_).

### Perspectives

The results of this study have some interesting perspectives for team sports training. Practitioners often use sprint time to assess performance during sprint training. There is an interest in quantifying the mechanical production of power, force, and velocity in sprinting, with or without fatigability conditions, to improve training programming. For both groups, coaches could prioritize the training of horizontal force at high velocity under fatigue conditions, as this ability tends to be the most affected. They could also incorporate training on mechanical effectiveness as this is a determinant of repeated sprint performance in adolescents [4], which is essential in team sports [40]. In addition, this training programme could also be of interest since children need to develop their motor skills at low and high velocities during their neural development. Finally, a better understanding of the optimal workload required to achieve a desired level of fatigability could also lead to more accurate programming of training loads.

## Supporting information

S1 Dataset (XLSX)

S1 Table Degrees of freedom and test statistics for F-v and mechanical effectiveness parameters, sprint time performance, RPE and FI. *DF*, degrees of freedom*; F*, test statistic*; P*_max_, maximal power output; *F*_0_, theoretical maximal force; *v*_0_, theoretical maximum velocity; *S*_FV_, force-velocity slope relative to body mass; *RF*_max_, maximum value of ratio of force; *D*_RF_, decrement in ratio of force; RPE, rating of perceived exertion; *FI*, fatigue index.
(DOCX)
