# Supplementary material for: Effect of fatigability on sprint time performance and force-velocity profile according to maturity status in young rugby players
Source: PLoS One. 2025 Jan 15;20(1):e0316947. doi: 10.1371/journal.pone.0316947 (PMC11734967; doi:10.1371/journal.pone.0316947)
Supplement: S1 Table — DF, degrees of freedom; F, test statistic; Pmax, maximal power output; F0, theoretical maximal force; v0, theoretical maximum velocity; SFV, force-velocity slope relative to body mass; RFmax, maximum value of ratio of force; DRF, decrement in ratio of force; RPE, rating of perceived exertion; FI, fatigue index. (DOCX) [file pone.0316947.s002.docx]

|  | **Block** | | **Maturity status** | | **Block*Maturity status** | |
| --- | --- | --- | --- | --- | --- | --- |
| **Variables** | **DF** | **F** | **DF** | **F** | **DF** | **F** |
| *P*_max_ (%) | 6.0 | 17.7 | 6.0 | 0.7 | 6.0 | 0.2 |
| *F*_0_ (%) | 6.0 | 9.1 | 6.0 | 0.1 | 6.0 | 0.5 |
| *v_0_* (%) | 4.0 | 17.3 | 4.0 | 0.7 | 4.0 | 0.2 |
| *S*_FV_ (%) | 6.0 | 6.9 | 6.0 | 1.0 | 6.0 | 0.7 |
| *RF*_max_ (%) | 6.0 | 12.3 | 6.0 | 0.5 | 6.0 | 0.2 |
| *D*_RF_ (%) | 6.0 | 6.8 | 6.0 | 1.0 | 6.0 | 0.6 |
| 30-meter sprint time (%) | 2.9 | 17.2 | 2.9 | 0.4 | 2.9 | 0.1 |
| RPE | 2.2 | 111.0 | 2.2 | 1.0 | 2.2 | 0.4 |
| FI (%) | 1.3 | 26.2 | 0.2 | 26.2 | 1.3 | 0.4 |
